# Presenting features of idiopathic versus secondary restless legs syndrome in pregnancy

**Published:** 2014-10-06

**Authors:** Shahnaz Miri, Mohammad Rohani, Mansoureh Vahdat, Maryam Kashanian, Elaheh Sariri, Babak Zamani, Gholam Ali Shahidi

**Affiliations:** 1 Department of Neurology, School of Medicine, Downstate Medical Center, State University of New York, Brooklyn, New York, NY, USA; 2 Department of Neurology, School of Medicine, Rasool Akram Teaching Hospital, Iran University of Medical Sciences, Tehran, Iran; 3 Department of Obstetrics & Gynecology, School of Medicine, Rasool Akram Teaching Hospital, Iran University of Medical Sciences, Tehran, Iran; 4 Department of Obstetrics & Gynecology, School of Medicine, Akbar Abadi Teaching Hospital, Iran University of Medical Sciences, Tehran, Iran

**Keywords:** Restless Legs Syndrome, Idiopathic and Secondary Form, Pregnancy, Sleep

## Abstract

**Background: **Restless legs syndrome (RLS) is the most common movement disorder in pregnancy, which can be idiopathic or secondary. There are limited comparative data regarding these two forms of RLS. The aim of this study was to compare clinical features of idiopathic and secondary RLS in pregnant women.

**Methods:** Over a period of 3 months, 443 women who admitted for delivery in two clinical centers were screened for RLS using four diagnostic criteria of the international RLS study group. A total of 79 subjects diagnosed with RLS were consecutively enrolled in the present study. All of them were interviewed for medical history and complaints during pregnancy and responded to self-administer international RLS rating scale.

**Results: **Ten subjects (12.9%) out of 79 pregnant women with RLS had idiopathic form, and their mean age was significantly higher than patients with secondary RLS (30.6 ± 7.3 years vs. 26.4 ± 4.6 years, P = 0.0260). Compared with women with secondary RLS, sleep duration in pregnancy was significantly decreased in idiopathic RLS group (P = 0.0460), whereas RLS severity score was similar in both groups. No significant difference was observed between the two groups in terms of other sleep complaints, the positive family history of RLS, parity, duration of pregnancy, or frequency of cesarean section (P > 0.0500).

**Conclusion: **Idiopathic and secondary RLS have relatively similar courses and features during pregnancy. However, the idiopathic form may have more negative impact on sleep in pregnancy. Careful screening and effective treatment of idiopathic RLS before pregnancy is recommended to limit these disturbances.

## Introduction

Restless legs syndrome (RLS) is a relatively common movement disorder characterized by an irresistible urge to move the legs, usually accompanied by an uncomfortable sensation within the legs, especially during inactivity. Symptoms worsen in the evening or at night and temporarily relieve with activity.^1^ RLS can be idiopathic, or it may develop secondary to a variety of medical conditions, including pregnancy, iron deficiency, end-stage renal disease, diabetes mellitus, rheumatoid arthritis, and neurological disorders.^2^

Pregnancy is considered as a major cause for secondary RLS, although up to 37% of pregnant women with RLS may have symptoms onset before the pregnancy.^1^^,^^3^ More than half of women with pre-existing RLS experience worsening of symptoms during pregnancy and their symptoms often present earlier than women with a secondary form.^4^^,^^5^ On the other hand, RLS can contribute to sleep disturbances and poor quality of sleep in pregnant women.^5^ It has been hypothesized that poor sleep in pregnancy contributes to increased risk of complications, such as intrauterine growth retardation, and preterm labor.^6^

To the best of our knowledge, little is known about the differences between idiopathic RLS in pregnancy and the secondary form regarding the clinical features and outcomes. The aim of the present study was to determine the prevalence of idiopathic form of RLS in pregnant women and to compare presenting features in idiopathic versus secondary RLS during pregnancy.

## Materials and Methods 

This cross-sectional study was conducted in obstetrics wards of Rasool-Akram and Akbarabadi Teaching Hospitals, Tehran, Iran, between the months of January 2011 and April 2011. The study was affiliated with the Iran University of Medical Sciences and was approved by Ethical Committee of the University.

The study population included women who admitted to the postnatal ward of the mentioned hospitals. They were interviewed within 2 days after delivery. Woman who had underlying diseases such as diabetes mellitus, renal failure, and neurological disorders (all other secondary possible causes of RLS) or women with a complicated pregnancy (pre-eclampsia, eclampsia, and gestational diabetes mellitus) were excluded. All participants signed a written informed consent.

A total of 443 consecutive women were screened by the four standardized diagnostic criteria of international RLS study group (IRLSSG)^1^ and among them, 79 patients were identified to have RLS symptoms during pregnancy (positive answer to all four criteria). The RLS-positive subjects were enrolled in the study. According to the onset of the syndrome (before pregnancy or during pregnancy) and underlying causes, patients were divided into two groups: patients with idiopathic RLS (10 cases) or patients with RLS secondary to pregnancy (69 cases).

The subjects underwent a structured face to face interview and data about age, parity, family history of RLS were obtained. Furthermore, sleep complaints such as insomnia (having experience of insomnia more than 2 times/week during the last month) and excessive daytime somnolence (experiencing sleepiness more than 2 times/week during the last month), and early morning awaking was investigated. The duration of sleep and latency before sleep were recorded.

Information about pregnancy duration, occurrence of preterm labor, and surgical delivery were collected. Patients were also asked to report the severity of symptoms by the IRLSSG severity rating scale.^7^

Data were analyzed using SPSS for Windows 11.0 (SPSS Inc., Chicago, IL, USA). Qualitative variables were presented as a percentage and means ± standard deviation used to demonstrate quantitative variables. Univariate analysis was performed to evaluate any relationship with RLS form, using Student’s t-test and χ^2^ or Fischer exact tests. A probability level below 0.05 was considered as statistically significant.

## Results

Diagnosis of RLS was made in 79 out of 443 pregnant women (17.8%). Ten patients (12.9%) had onset of RLS symptoms prior to pregnancy and had no associated disease that can induce RLS, including anemia, renal failure, diabetes mellitus, and other neurological disorder (idiopathic form). 

Mean duration of symptoms onset was 17.8 ± 16.3 months in idiopathic RLS patients, which was significantly longer than patients with secondary form (3.1 ± 2.1 months; P = 0.0001). Nevertheless, RLS severity according to IRLSSG rating scale did not demonstrate notable difference between idiopathic and pregnancy-related RLS patients (16.0 ± 4.8 vs. 15.5 ± 4.7, respectively; P = 0.7800) (Table 1).

Patients with secondary RLS had significantly lower age than patients with primary RLS (26.4 ± 4.6 years and 30.6 ± 7.3 respectively; P = 0.0100). However, there was no considerable difference between two groups regarding the parity and family history of RLS.

The univariate analysis exhibited a significant relationship between idiopathic RLS and shorter duration of sleep in pregnancy (P = 0.0400). However, no significant difference was observed between the idiopathic RLS and secondary RLS patients in terms of other sleep disturbances, including increased sleep latency, insomnia, early morning awaking, and daytime somnolence (P > 0.0500).

The mean pregnancy duration of idiopathic and secondary RLS was 38.5 ± 1.0 weeks and 38.2 ± 2.3 weeks, respectively, with no significant difference between two groups (P = 0.7000). Preterm labor was non-significantly higher in women with secondary RLS than with primary type (18.8% vs. 0.0%, respectively, P = 0.1300). The rate of operative delivery was not clearly different between the pregnant women with idiopathic and secondary RLS (P = 0.4000).

**Table 1:** Comparison on idiopathic and secondary restless legs syndrome in pregnant women

**Variables**	**All RLS patients ** **(n = 79)**	**RLS onset before ** **pregnancy (n = 10)**	**RLS onset in ** **pregnancy (n = 69)**	**P**
Age (years)	26.9 ± 5.1	30.6 ± 7.3	26.4 ± 4.6	0.0160*
Nulliparity, n (%)	26 (32.9)	1 (11.1)	25 (36.2)	0.0990
Family history of RLS, n (%)	15 (19.0)	3 (30.0)	12 (17.3)	0.3420
RLS duration (months)	5.0 ± 7.6	17.8 ± 16.3	3.1 ± 2.1	0.0001*
RLS severity score	15.6 ± 4.7	16.0 ± 4.8	15.5 ± 4.7	0.7890
Sleep latency (min)	57.2 ± 45.7	66.0 ± 57.3	56.0 ± 44.1	0.5240
Sleep duration (h)	7.1 ± 2.1	5.9 ± 2.5	7.3 ± 2.0	0.0460*
Insomnia, n (%)	35 (44.3)	6 (60.0)	29 (42.0)	0.2330
Early awaking, n (%)	37 (46.8)	6 (60.0)	31 (44.9)	0.3720
Daytime somnolence, n (%)	37 (46.8)	3 (30.0)	34 (49.2)	0.2130
Pregnancy duration (weeks), n (%)	38.2 ± 2.2	38.5 ± 1.0	38.2 ± 2.3	0.7000
Preterm labor, n (%)	13 (16.4)	0 (0.0)	13 (18.8)	0.1330
Delivery				
NVD, n (%)	33 (41.7)	5 (50.0)	28 (40.5)	0.4080
CS, n (%)	46 (58.2)	5 (50.0)	41 (59.4)	

RLS: Restless legs syndrome; NVD: Natural vaginal delivery; CS: Cesarean section;

* Statistically significant difference

## Discussion

RLS has been described as the most common movement disorder during pregnancy.^8^ The syndrome can start after pregnancy (secondary form) or it may be present before pregnancy (idiopathic form).^2^ In the general population, idiopathic form is the most common type of the RLS.^9^ However, the secondary form is more prevalent among pregnant women. Consistent with the study of Uglane et al. 82.3% (70 of 85) of pregnant women with RLS, developed symptoms secondary to pregnancy.^10^ As well, according to the study of Lee et al. 63% of pregnant women with RLS had not symptoms before the pregnancy.^3^ In accordance with prior studies, our findings showed that 12.9% of the pregnant women with RLS had idiopathic form of the disease and 87.3% had secondary form.

RLS usually presents during the second or third trimester.^8^ Nevertheless, symptoms of idiopathic RLS mostly present before the third trimester.^5^ As we observed, the RLS onset was approximately 3 months before delivery in secondary RLS. However, the duration of symptoms was longer in those with idiopathic RLS.

It has been assumed that severity of RLS symptoms may worsen as the pregnancy progresses, with the highest degree in the third trimester.^11^ In addition, the probability of worsening symptoms during pregnancy is higher in women with pre-existing RLS.^5^ Furthermore, Manconi et al. reported in his large cohort of RLS in pregnancy, 61% of subjects affected by a pre-existing RLS experienced symptomatic aggravation during pregnancy.^4^ Therefore, it is possible that women with idiopathic RLS who had onset before pregnancy have more severe symptoms of RLS. However, according to our study, severity of RLS at the end of pregnancy was relatively equal among idiopathic and pregnancy-related RLS patients. Since we did not have any information about the severity of symptoms at onset of the disorder, we cannot discuss about the course of RLS severity among pregnant women with idiopathic or secondary RLS. On the other hand, because most of the cases had moderate symptoms at the end of pregnancy, we can conclude that there was not significant different between two groups in term of the course of RLS during pregnancy.

RLS may be associated with longer sleep latency and complaints of insomnia.^12^ In our study, idiopathic RLS patients had shorter duration of sleep than secondary form. However, the prevalence of other sleep disturbances, including increased sleep latency, insomnia, early morning awaking, and daytime somnolence were not different between two groups. These results show a slight different between the groups that are just attributed to sleep duration. Shorter duration of sleep in the idiopathic group may be due to longer duration of RLS and sleep complaints in these patients. Sleep disruption can negatively affect activities of daily living.^13^ This may lead to poorer outcomes in pregnant women. Therefore, we compared two groups from this point of view.

The mean pregnancy duration and the prevalence of preterm labor were similar in both groups, and it was comparable with pregnant women without RLS. The rate of operative delivery was not clearly different between the pregnant women with idiopathic and secondary RLS. Thus, pregnancy outcome is similar in both groups.

One limitation of the study was small sample size, in addition to the lack of the control group. Further investigations are needed to demonstrate especial features of RLS secondary to pregnancy.

## Conclusion

There are no significant differences between clinical features and outcomes of RLS among pregnant women with idiopathic or secondary form of the syndrome. Nevertheless, due to annoying symptoms of RLS in pregnant women, especially sleep disturbances, it is preferable to detect and treat women with idiopathic RLS before pregnancy.

## Notes

**How to cite this article: **Miri Sh, Rohani M, Vahdat M, Kashanian M, Sariri E, Zamani B, et al. Presenting features of idiopathic versus secondary restless legs syndrome in pregnancy. Iran J Neurol 2014; 13(4): 241-4.
